# G-actin guides p53 nuclear transport: potential contribution of monomeric actin in altered localization of mutant p53

**DOI:** 10.1038/srep32626

**Published:** 2016-09-07

**Authors:** Taniya Saha, Deblina Guha, Argha Manna, Abir Kumar Panda, Jyotsna Bhat, Subhrangsu Chatterjee, Gaurisankar Sa

**Affiliations:** 1 Division of Molecular Medicine, Bose Institute, P-1/12, CIT Scheme VII M, Kolkata-700054, India; 2 Division of Biophysics, Bose Institute, P-1/12, CIT Scheme VII M, Kolkata-700054, India

## Abstract

p53 preserves genomic integrity by restricting anomaly at the gene level. Till date, limited information is available for cytosol to nuclear shuttling of p53; except microtubule-based trafficking route, which utilizes minus-end directed motor dynein. The present study suggests that monomeric actin (G-actin) guides p53 traffic towards the nucleus. Histidine-tag pull-down assay using purified p53(1–393)-His and G-actin confirms direct physical association between p53 and monomeric G-actin. Co-immunoprecipitation data supports the same. Confocal imaging explores intense perinuclear colocalization between p53 and G-actin. To address atomistic details of the complex, constraint-based docked model of p53:G-actin complex was generated based on crystal structures. MD simulation reveals that p53 DNA-binding domain arrests very well the G-actin protein. Docking benchmark studies have been carried out for a known crystal structure, *1YCS* (complex between p53DBD and BP2), which validates the docking protocol we adopted. Co-immunoprecipitation study using “hot-spot” p53 mutants suggested reduced G-actin association with cancer-associated p53 conformational mutants (R175H and R249S). Considering these findings, we hypothesized that point mutation in p53 structure, which diminishes p53:G-actin complexation results in mutant p53 altered subcellular localization. Our model suggests p53Arg249 form polar-contact with Arg357 of G-actin, which upon mutation, destabilizes p53:G-actin interaction and results in cytoplasmic retention of p53R249S.

p53, the guardian of genome, executes its tumor suppressor function through maintenance of the genetic integrity, cell-cycle machinery, apoptosis and DNA repair^1^. In order to check genetic errors, p53 accumulates in the nucleus in response to cellular stress like DNA damage, hypoxia, and nucleotide deprivation^2^. Once p53 is transported into the nucleus, it trans-activates its target genes, involved either in cell-cycle arrest (e.g., p21, 14-3-3) or apoptosis (e.g., BAX, PUMA, NOXA)^3^. p53 functions as a homo-tetramer. Each monomer of p53 (393 amino acid long) consists of an intrinsically disordered amino-terminal trans-activation domain (Met1-Asp42), a proline-rich domain (Asp61-Ser94), a DNA-binding domain (Thr102-Lys292) and an unstructured carboxy-terminal domain (Pro301-Asp393) containing a tetramerization domain (Asp324-Ala355)^4^. The transit of p53 from cytosolic environment to nucleus is an essential event as it is the site where p53 functions as a transcription factor. Microtubules and its associated ‘−’ end-directed motor protein dynein have been proposed to participate in nuclear transport of p53 following DNA damage^5^. In this context, a recent report by Wang *et al*. suggests that polymerization of actin negatively regulates the nuclear import of p53^6^. This preliminary finding hints that parallel to microtubule-based p53 traffic, some other mode of transport might also exist for p53 that involve another cytoskeletal protein, actin.

Actin participates in diverse protein-protein interaction and plays multi-dimensional cellular functions; ranging from cell motility, maintenance of cell shape, cell polarity to regulation of transcription. Actin exists in 2 forms- monomeric G-actin and polymeric F-actin. Recently, an actin-dependent mechanism for long-range transport of vesicles has been reported^7^. Actin regulates the subcellular localization of “junction mediating and regulatory protein”, a transcriptional co-activator^8^. It was reported that an actin-binding RPEL motif present in “myocardin-related transcription factor”, regulates its nucleo-cytoplasmic shuttling^9^. Notably, wild-type p53 has been found to be very closely associated with the cytoplasmic actin filaments at the time of DNA synthesis^10^. Overall these evidence suggest that transport of p53 might require its association with actin. The objective of the present work is to determine the contribution of specific structural forms of actin (monomeric/fibrous), which might be involved in p53 nuclear import.

Alteration in the protein structure in course of mutation has been recognized as an important factor that affects p53 localization. Mutation in its DNA-binding domain (DBD) is a frequent event in cancer which contributes to the proliferation and differentiation of the neoplastic population. According to IARC TP53 mutation database, single point mutations in p53DBD accounts for more than 95% of the reported malignant mutations^11^. Six amino acid residues in p53DBD have been mapped, having the highest frequency of mutation, popularly known as sites of “hot-spot” mutations^11^. The core domain p53 mutants can be distinguished into–contact and structural mutants. In response to genotoxic stress, weakly destabilized p53 contact-mutants (R273H and R248W), predominantly show nuclear distribution. On the contrary, structural-mutants, R175H, R248Q, R249S, R250L, and R110P typically show perinuclear localization of protein aggregates^12^.

Our study reveals monomeric G-actin directs p53 traffic towards the nucleus through protein-protein interaction. A characteristic perinuclear colocalization between p53 and G-actin is evident in response to genotoxic stress. Considering p53 ‘hot-spot’ mutants, our study suggests that structural mutants show reduced degree of association with G-actin, resulting in altered localization of mutant p53. Based on the experimental information, a hypothetical model of p53:G-actin complexation has been derived using molecular docking and MD simulation studies. Our study explores p53Arg249 to be situated at the p53:G-actin interface, which upon mutation into Serine (p53R249S) abrogates the protein-protein complex, and results in its cytosolic sequestration.

## Results

### Tracing p53 nuclear translocation using actin stabilizing/depolymerizing drugs

**Figure 1:**
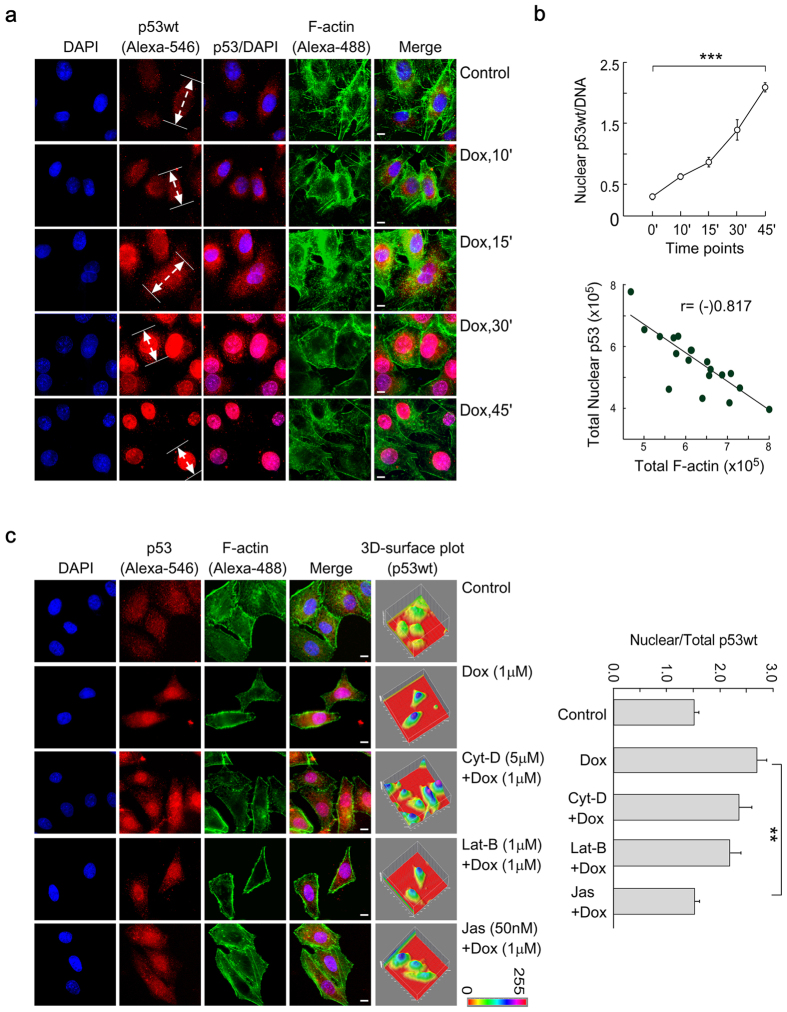
p53 nuclear translocation depends on the F-/G-actin dynamicity. (**a**) Wild-type p53-expressing cells were treated with 1 μM doxorubicin for different time intervals. Wild-type p53 was stained with the Alexa-546-tagged anti-p53 antibody (DO-1). F-actin and nucleus were stained with Alexa-488-tagged phalloidin and DAPI respectively. Scale bar: 10 μm. (**b**) Nuclear p53 was quantitated by quantitative fluorescent imaging and normalized with nuclear DNA content (*upper panel*). Scatter plot of total cellular F-actin and nuclear p53, generated at different time points after Dox treatment (*bottom panel*). The solid line shows the best line fitted to the data based on the simple regression model. The correlation coefficient (r) was mentioned in the graph. (**c**) The degree of p53 nuclear translocation at different drugs-treated conditions; scale bar: 10 μm. 3D-surface plots are shown in the right panel. Nuclear/total p53 was quantitated by quantitative fluorescent imaging and has been plotted for the above experimental conditions (*right*). Values are mean ± SEM of five independent experiments in each case or representative of typical experiment.

Tumor suppressor p53 is a short-lived protein, where genotoxic stress is responsible for increasing its half-life^13^. We used doxorubicin (Dox) to generate cellular stress to provoke the accumulation of p53 in the nucleus^14^. With time, Dox (1 μM) induced p53 level, and the majority of the p53 pool was transported into the nucleus (Fig. 1a). Analysis of plot profiles for p53 distribution revealed the same (Suppl. Fig. 1a). This inference is conceivable from the quantitative image analysis data where total nuclear p53 level was normalized with the DNA content (Fig. 1b, *upper panel*). There was a significant negative correlation [r = (−)0.817] between p53 nuclear import and the level of total F-actin in Dox-treated cells (Fig. 1b, *bottom panel*).

Since p53 nuclear transport was increased with the concomitant decrease in F-actin level, we next intended to test whether actin disruption has any direct effect on p53 transport. Different drugs have been chosen that alter the cellular actin dynamics; e.g., Cytochalasin-D (Cyt-D) - inhibits actin polymerization; Latrunculin-B (Lat-B) - impairs addition of actin subunits to the filament ends, and Jasplakinolide (Jas) - increases actin nucleation. The altered actin structure and respective 3D-surface plots in response to Cyt-D, Lat-B and Jas have been shown in Supple. Fig. 1b. In cases of Cyt-D and Lat-B treatment, fibrous actin became depolymerized as shown by the punctate phalloidin staining (Fig. 1c). When Cyt-D-/Lat-B-primed cells were treated with Dox, no significant alterations in p53 nuclear-trafficking were observed (Fig. 1c). Interestingly, Jas, that stabilized F-actin, significantly abolished Dox-induced p53 nuclear import (Fig. 1c). 3D-surface plots in Fig. 1c showed the distribution of p53 at different stress-conditions. Importantly, when the F-actin/G-actin ratio is tilted towards F-actin, a reduced nuclear accumulation of p53 is observed. This indicates that probably G-actin is playing an important role for p53-nuclear translocation event.

### Fluctuation in F/G-actin ratio results in altered p53 nuclear transport

Next, we sought to study the F/G-actin ratio in different actin perturbing drugs-treated conditions; to correlate the data with the degree of p53 nuclear import. For this purpose, A549 cells were treated with only Dox or in combination with Cyt-D/ Lat-B/ Jas. It was observed that in response to Dox, there was an increase in the level of G-actin, which showed an intense distribution in the perinuclear region (Supple. Fig. S2a,b). Plot profile analysis and mean gray value of G-actin distribution in control/Dox-treated cells indicate the same. In HCT116 cells, an increase of G-actin expression was also noticed in presence of Dox (Supple. Fig. S2c). These results are in accordance with the previous report by Litwiniec *et al*., which suggested perinuclear staining pattern of G-actin upon treatment with etoposide^15^.

**Figure 2:**
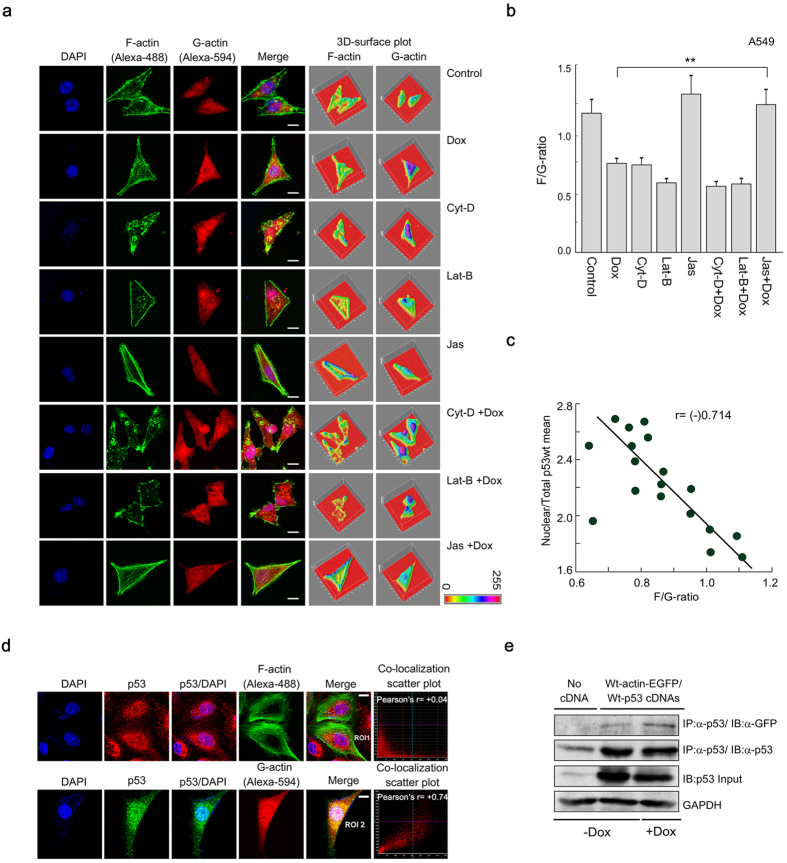
Nuclear translocation of p53 is negatively-correlated with the shift in F/G-actin ratio. (**a**) Confocal images showing the distribution of F- and G-actin in different drugs-treated conditions. F- and G-actin were stained with Alexa-488-tagged phalloidin and Alexa-594-tagged DnaSe1 respectively. Nucleus was stained with DAPI. Magnification 60×. 3D-surafce plots show the respective distribution of F- and G-actin. Scale bar: 10 μm. (**b**) Bar diagram of F-/G-actin ratios in the different drugs-treated conditions in A549 cells. (**c**) Scatter plot showing negative-relationship between nuclear/total p53 and F/G ratios. The solid line shows the best line fitted to the data based on the simple regression model. The correlation coefficient (r) was mentioned in the graph. (**d**) Colocalization between p53 and F-actin/G-actin in A549 cells. p53 was stained with Alexa-546/-488-tagged p53 (DO-1) antibody. F-/G-actin were directly stained with Alexa-488-tagged phalloidin or Alexa-594-tagged DnaSe1 respectively. Nucleus was stained with DAPI. Image magnification: 60×. Scale bar: 10 μm. Pearson’s colocalization correlation(r) between red and green channels is presented in colocalization scatter plots. (**e**) A549 cells were co-transfected with EFGP-tagged wild-type actin and wild-type p53. p53-associated proteins were pulled-down with p53 antibody and Western blotted with anti-GFP and anti-p53 antibodies. GAPDH was used as internal loading control. Data are representative or as the mean ± SEM and are the cumulative results of five independent experiments (n = 25), *p < 0.05, **p < 0.01, ***p < 0.001.

Earlier report suggests that actin stabilization retards nuclear transport of p53^6^. This study indicates that alteration of F/G-actin ratio might influence the amount of p53 transported into the nucleus. Therefore we studied the alteration in the F/G-actin ratio in response to actin disrupting drugs, in presence or absence of Dox. The distribution of both F- and G-actin in different drug-treated condition has been studied with the help of confocal imaging, based on which a quantitative relationship between nuclear/total p53 mean and F/G-ratio has been generated (Fig. 2c). There is an increase in G-actin concentration in Dox-treated cells, which is reflected in the corresponding fall of F/G-actin ratio (Fig. 2a,b). In response to Cyt-D + Dox, fibrous actin was totally disrupted and level of G-actin was increased. Likewise, in Lat-B + Dox condition, the net G-actin pool was increased because the F-actin polymerization process slowed down. However, in the case of Jas + Dox treatment, F/G-actin ratio increased as an assembly of actin filaments was prompted. From these observations, it can be suggested that due to Cyt-D/Lat-B pre-treatment, the F/G-ratio dropped (Fig. 2b). Since the level of G-actin increased due to Cyt-D/Lat-B treatment (Fig. 2a,b), p53 was transported to the nucleus (Refer to Fig. 1c). This finding is in accordance with the report that actin filament disruption results in p53 activation^16^. However, the unavailability of monomeric G-actin, in presence Jas (Fig. 2a,b) is believed to retard the nuclear shuttling of p53 even in the presence of Dox (Refer to Fig. 1c). 3D-surface plots reflect a positive correlation exists with respect to the distribution of p53 and G-actin in different conditions (Supple. Fig. S2d). This explains when the level of G-actin falls, p53 nuclear accumulation is less; whereas when the G-actin level increases in Dox-treated condition, p53 translocates to the nucleus. Graphical representation of F/G ratio reveals that there is a significant change (P < 0.01) in the F/G ratio for Jas+Dox condition compared to Dox alone condition (Fig. 2b). An inverse correlation-ship was observed between nuclear p53 vs F/G ratio in actin-disrupted conditions [r = (−)0.714] (Fig. 2c).

Co-transfection of A549 cells with wild-type p53 and EGFP-tagged wild-type actin revealed interaction between these two molecules (Fig. 2e). To find out the type of actin involved in such interaction we performed colocalization experiments with actin-specific probes. Wild-type p53 expressing A549 cells showed a significant degree of positive colocalization correlation between p53 and G-actin (Fig. 2d, *bottom panel*). On the contrary, p53 did not show significant positive Pearson’s colocalization correlation with F-actin (Fig. 2d, *upper panel*). Pearson’s r obtained through quantitative image analysis was found to be significant (p < 0.005) for p53 and G-actin (0.80), compared to the values found in the case of p53 and F-actin (0.1), indicated in Supple. Fig. S3a.

### Wild-type p53 directly interacts with G-actin and shows distinct perinuclear colocalization

**Figure 3:**
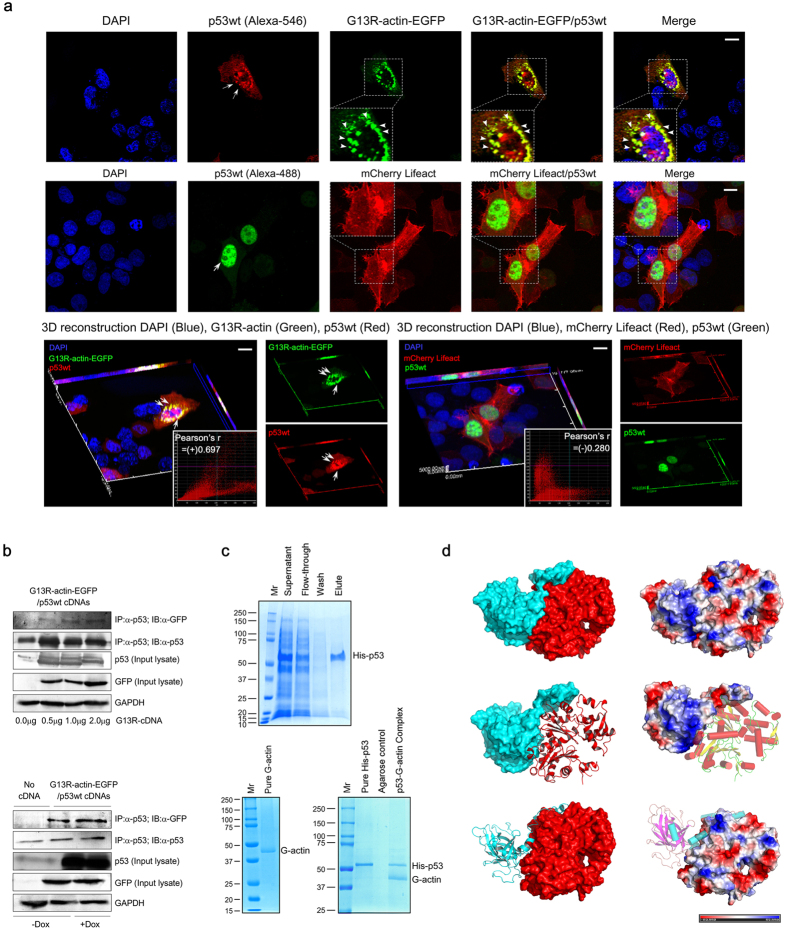
Interaction of wild-type p53 with the polymerization-deficient G13R-actin mutant. (**a**) Colocalization of wild-type p53 with EGFP-tagged G13R-actin in the perinucleus. Over-expressed wild-type p53 was stained with the Alexa-546-tagged anti-p53 antibody (*upper panels*). Wild-type p53 (stained with Alexa-488-tagged anti-p53 antibody) did not show any positive colocalization with F-actin (stained with mCherry-Lifeact) (*middle panels*). The selected ROIs have been magnified in the inset images where the pixels showing intense colocalization are marked with arrowheads. Image magnification: 60×. Scale bar: 10 μm. Three-dimensional (3D) confocal microscopy revealed punctated colocalized yellow pixels of EGFP-G13R-actin and wild-type p53, shown by arrowheads. 3D-reconstruction image shows no correlation between mCherry Lifeact and wild-type p53. Pearson’s colocalization correlation (r) between red and green channels is presented in colocalization scatter plots (*inset images, lower panels*). (**b**) Physical association between wild-type p53 and G-actin, at increasing concentrations (0.1, 0.5, 1.0, 2.0 μg) of G13R-actin-EGFP cDNA, indicated in the *upper panel*. Association of wild-type p53 and G-actin in control and Dox-treated condition were presented in the *lower panel*. GAPDH was used as the internal loading control. Data are representative three independent experiments. (**c**) His pull-down assay shows purified p53(1–393)His and purified monomeric G-actin directly interacts *in vitro*. The purified His6-p53 protein has been detected in the Ni-NTA column ‘elute’ fraction, *upper panel*. Purification of G-actin (*lower panel, left*). Purified His6-p53 (400 μg) was incubated with 170 μg of monomeric G-actin in Ni-NTA column. Coomassie staining reveals the position of p53His and G-actin in ‘bait-prey’ complex (*lower panel, right)*. (**d**) Post-simulation p53:G-actin dynamic complex, in surface/electrostatics (*upper panel*), p53 in surface/electrostatics and G-actin in cartoon/secondary structure (*middle panel*), p53 in cartoon/secondary structure and G-actin in surface/electrostatics (*lower panel*). p53 and G-actin have been shown in cyan and red color respectively.

Since our preliminary findings suggest that p53 colocalized with monomeric G-actin, next our intention was to study whether it involves protein-protein interaction. For this purpose, we selectively expressed non-polymerizable actin mutant (G13R-actin) to exogenously produce GFP-chimeric G-actin, defective in forming fibrous actin^17^. Since the mutation occurs at the nucleotide-binding pocket, it interferes with ATP-binding and keeps the protein in monomeric state. Confocal imaging revealed that G13R-actin strongly colocalized with wild-type p53 in the perinuclear region and p53 molecules translocate to the nucleus (Fig. 3a). 3D-reconstruction images distinctly revealed the merged pixels in yellow, where these two proteins colocalized. Quantitative image analysis revealed a high-level of positive colocalization correlation [Pearson’s r = (+)0.697] (Fig. 3a, *lower left*). Colocalized pixels showed a distribution along the linear regression line of the scatter plot.

Physical interaction between p53 and G-actin was validated by co-immunoprecipitation study when GFP-tagged G13R-actin was pulled down by p53 antibody (DO-1). Co-transfection of varying concentrations of wild-type p53 and G13R-actin-EGFP revealed that the degree of physical interaction between these two proteins increased with the higher amount of transfected cDNA (Fig. 3b, *upper panel*). Dox treatment further augmented such interaction (Fig. 3b, *lower panel*). With this observation, we were curious to identify whether this interaction is a direct protein-protein interaction or is mediated *via* any other protein. In order to establish this *in vitro,* histidine pull-down assay was performed using purified His6-p53 and G-actin^18^. Ni-NTA-beads bound His6-p53 protein was purified using gravity flow column (Fig. 3c, *upper panel*). The purified His6-p53 protein was incubated with purified G-actin and the bound proteins were analyzed by SDS-PAGE followed by coomassie staining, which distinctly revealed bands for both His6-p53 and G-actin (Fig. 3c, *lower right panel*).

G13R-actin, which codes for monomeric actin population, colocalized and directly interacts with p53. With this crucial finding, we tried to address the events in an experimental condition where the G13R-actin mutant is substituted with a plasmid coding for filamentous actin. Hence, we involved Lifeact in our study to explore the relative correlation of distribution between filamentous actin with p53. Lifeact is 17 amino acid-long peptide tagged with m-Cherry, which selectively binds to F-actin (Supple. Fig. S3a) and fluoresce^19^. p53 did not show any positive colocalization correlation [Pearson’s r = (−)0.280] with F-actin, when lifeact was used (Fig. 3a, *lower right panel*), though wild-type p53 shuttled inside the nucleus. Colocalization correlation value for G13R actin-p53 wild-type pair is significantly higher compared to Lifeact-p53 wild-type pair (p < 0.005) (Supple. Fig. S3a).

To get the atomistic insight of p53:G-actin interaction, we undertook protein-protein docking approach using crystal structures (*PDB code: 4MZR for p53, fragment: 94*–*356 and 3HBT for G-actin)* (Supple. Fig. S3c). Here we mainly used two different docking algorithms, ZDOCK version 3.0.2^20^ and Schrödinger’s protein-protein docking module PIPER^21^. To delineate the robustness of our derived p53:G-actin model, docking benchmark studies using a known crystal structure (*PDB code: 1YCS*) was carried out. *1YCS* is the crystal structure of the complex between p53DBD and BP2 protein^22^. Our objective was to reproduce a known crystal structure as a control for docking calculation, which will validate the docking algorithm we have adopted. Both the docking software were used to reproduce the crystal structure *1YCS* as well as to obtain the p53:G-actin complex. Importantly, ZDOCK has been reported to successfully reproduce protein-protein docking benchmark of 176 test cases (http://zdock.umassmed.edu/help.html). Furthermore, literature suggests that knowledge of interface residues constrain the initial search space of docking software, which considerably improves the accuracy of protein-protein docking^23^. For this purpose, during the docking calculation between p53DBD and BP2, Arg248 (p53) was selectively mentioned as a contacting residue in the interface, as derived from analysis of *1YCS*. With this information, the crystal structure was reproduced as the top models (Model-1, RMSD = 1.330 Å, and Model-3, RMSD = 1.312 Å) among the ZDOCK predictions (Supple. Fig. S4). Parallel to this, when another amino acid constraint (Met243) was used for docking calculation; the crystal structure was again reproduced (Model-1, RMSD = 1.330 Å, and Model-4, RMSD = 1.312 Å). Even in the case of blind docking, where no constraint was used, the crystal structure was reproduced (Model-3, RMSD = 1.329 Å). In case of *1YCS*, all atoms RMSD of reproduced structures were found to be within 1.5 Å. Moreover, when the top 5 models were superimposed upon *1YCS*, all the predicted models showed similar docked poses with that of the crystal structure (data not shown). Schrödinger’s protein-protein docking module PIPER was also found to be successful in reproducing *1YCS* for blind/constraint-driven docking calculation. Hence both the docking modules successfully reproduced *1YCS,* irrespective of no constraint, or single point constraint. According to the energy-scoring function, these reproduced structures attained top ranking positions and showed minimum RMSD values as compared to *1YCS* (Supple. Fig. S4). These observations provide the docking benchmark study for 2 different docking approaches for a crystal structure and validate the accuracy of the docking protocols in predicting near to correct complexes. Since the co-immunoprecipitation data from our mutational studies suggests that the association between p53:G-actin is reduced in case of p53R249S mutant (Reference: Fig. 5c & Supple. Fig. S3d), Arg249 was considered as a residue participating in p53:G-actin interaction. Here we used Arg249 (p53) as a constraint for p53:G-actin docking calculation (similar to Arg248/Met243 constraint in p53 and BP2 docking calculation). Our observation was that Model-1, -2 and -4 (ZDOCK) exhibited similar structures. Using the same parameters, when this docking calculation was done with PIPER, similar docked pose (Model-5) was derived (Suppl. Fig. S4). Similar docked-poses for both p53:BP2 and p53:G-actin were derived using another protein-protein docking algorithm ClusPro 2.0^24^ (Suppl. Fig. S5). Importantly, the same docked pose for p53:G-actin complexation was produced with various docking approaches, and for ZDOCK the pose appeared for 3 times among the top 5 models (Suppl. Fig. S4, *third panel*). By the ranking of the model and the fact whether it appears multiple times among the top 5 models with varied docking algorithms, we selected Model-1 (ZDOCK) and carried out all atoms Molecular Dynamics (MD) Simulation (AMBER14) in an implicit solvent for 50 ns. Details of docking benchmark studies, docking calculation, and MD simulation have been included in the *Materials and Methods* section. Since the parameters were proved to be successful for reproducing a known crystal structure *1YCS,* with the help of different docking algorithms, our assumption is that the theoretically-derived p53:G-actin complex is most likely the probable structure of the real association. Post-simulation p53:G-actin model and the surface electrostatics of the complex are represented in Fig. 3d.

### Molecular interface in cross-linked actin dimer overlaps with p53:G-actin interface: basis of p53:G-actin interaction

**Figure 4:**
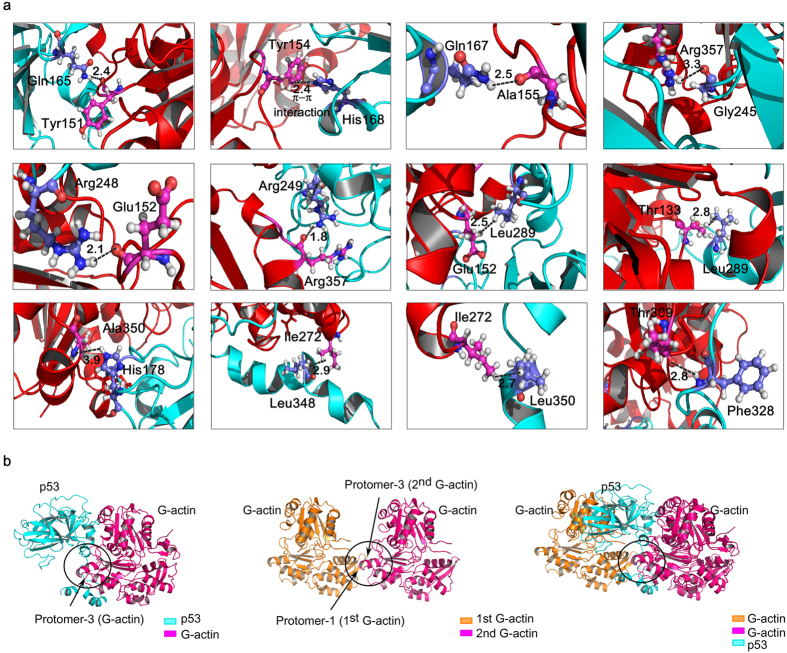
Theoretical understanding of p53:G-actin interaction from molecular docking and MD simulation studies. (**a**) Magnified images of amino acid contacts in p53:G-actin interaction in cartoon representation. The interacting amino acids are highlighted in sticks/spheres. Black dashed lines indicate the p53:G-actin interaction. Bond distances are included. (**b**) Structural superimposition shows a common interface of actin involved in actin:actin and p53:actin complexes. p53:G-actin docked complex has been shown in cartoon structure (cyan and purple color) in the *left panel*. Actin:actin dimer has been represented as cartoon structure (orange and purple color) in the *middle panel*. The interface of p53:G-actin and G-actin:G-actin complex has been marked with a circle. Superimposed structures of actin:actin and p53:G-actin docked model, *right panel*. Common interface of protomer-3 of G-actin involved in both the interaction has been marked with a circle.

Crucial amino acids contacts in p53:G-actin theoretical model (within 1.8 Å–3.9 Å) are shown in Fig. 4a. Amino acids Gln165, Gln167, His168, His178, Gly245, Arg248, Arg249, Leu289, Phe328, Leu348 and Leu350 of p53 form polar contacts with Tyr151, Tyr154, Ala155, Glu152, Thr133, Ile272, Ala350, Thr309 and Arg357 of G-actin respectively (Suppl. Table S1). The movie of MD simulation trajectories is included in the supplementary information (Suppl. Movie S1). The relative distance (>25 Å) of Gly13 (G-actin) from interacting interface of p53:G-actin model suggests that mutation at Gly13 does not interfere with p53:G-actin complexation (Suppl. Fig. S6a).

The theoretical understanding behind the differential affinity of p53 toward F-/G-actin was crucial to correlate the experimental findings. Inference for the same was identified with respect to the interface of G-actin involved in forming contacts with p53. Furthermore, the interface of monomeric actin involved in forming contacts within fibrous actin was analyzed. The crucial points of contacts in the actin-dimer crystal are mediated by protomer-1 of first G-actin and protomer-3 of second G-actin (*PDB code:* 2A5X)^25^. On the other hand, protomer-3 region of G-actin (indicated in Fig. 4b, *left panel*) was found to form contacts with p53 in the p53:G-actin dynamic model. Comparing these residues involved in making contacts, it is clear that the same interface (at protomer-3) of monomeric G-actin participates in both actin:actin and p53:G-actin interaction (Fig. 4b). Hence p53 is supposed to have poor affinity for fibrous-form of actin as the common interface for interaction is already engaged. A ribbon representation of this said event has been shown in Suppl. Fig. S6b.

### p53:G-actin interaction involving “hot-spot” mutants: basis of altered localization of mutant p53

Mutations in p53DBD have been reported to result in altered localization of the protein^26^,^27^. It is noteworthy to mention that fluctuation in thermodynamic stability of p53 arises due to single point mutations^4^. p53 contact mutants (R273H and R248W) retain same thermodynamic as well as kinetic stability^28^. On the other hand, p53 structural mutants (R175H and R249S) show a greater degree of thermodynamic instability and often show altered subcellular localization or cytoplasmic sequestration^12^. The assembly of mutant p53 into large aggregates in the cytosolic environment has been found to be consistent with an impaired p53 nuclear import^12^. Hence next our objective was to assess whether impaired binding to G-actin might explain the altered subcellular localization of the structural mutants. For this purpose, we selectively chose three ‘hot-spot’ point mutations (p53R273H-contact mutant; p53R175H and p53R249S-structural mutant). To satisfy our goal, we transiently over-expressed p53 mutants (R273H, R175H, and R249S) and G13R-actin-EGFP in p53-null H1299 cells, and studied the relative interaction of mutant p53 with G-actin.

**Figure 5:**
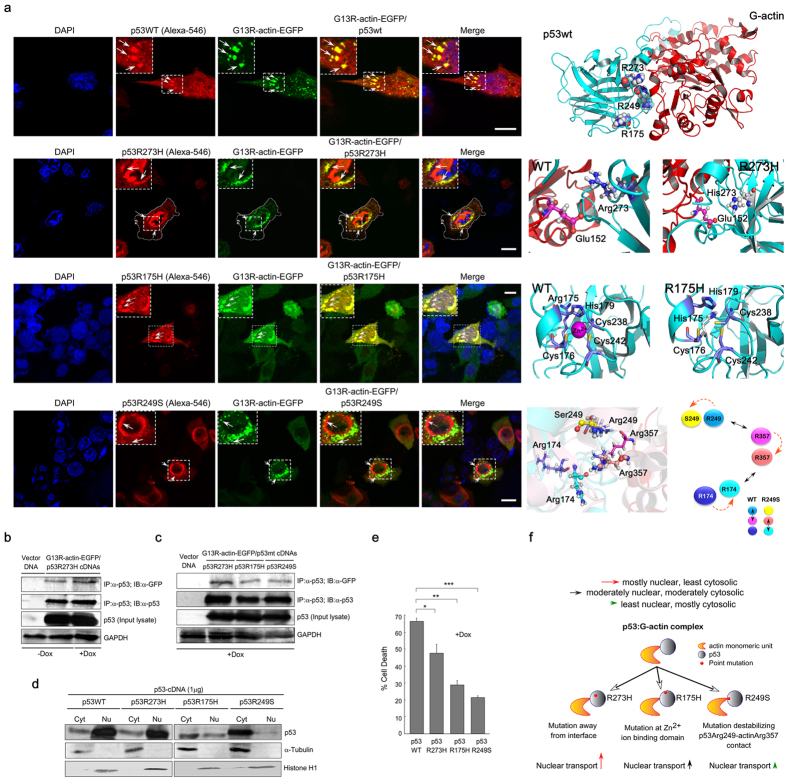
Degree of physical interaction between p53-mutants and G-actin dictates subcellular-localization of p53. (**a**) Colocalization of p53WT/p53R273H/p53R175H/p53R249S with G13R-actin-EGFP upon co-transfection in p53-null H1299 cells. Wild-type and mutant p53 were stained with Alexa-546-tagged anti-p53 antibody. Nucleus was stained with DAPI. The colocalized yellow pixels in the merge images are indicated with arrowheads. Image magnification: 60×. Scale bar: 10 μm. Cartoon representation of post-simulated p53:G-actin complex is shown in the *right panels*, showing the position of point mutations (p53) in each case (in spheres/sticks). Wild-type p53 is at the *left side* and mutant p53 is at the *right side* of *right panels*. For p53R249S, super-imposition of post-simulated p53:G-actin models (WT versus R249S) are shown in the bottom panels, showing the position of amino acid residues. A cartoon is shown depicting the amino acid shifts, where colored circles represent amino acid residues specified at left. Dotted-lines indicate the shifting of amino acid residues in R249S mutant. Double-headed arrows indicate the interaction between amino acids. Physical interaction between G13R-actin and p53-mutants, (**b**) p53R273H and (**c**) p53R273H/p53R175H/p53R249S, were performed by co-immunoprecipitation (with anti-p53 antibody) and Western blot (with anti-GFP antibody) assays. GAPDH has been used as the internal loading control. (**d**) Cytosolic and nuclear lysates from ectopically wild-type/mutant p53-expressing HCT116 (p53^−/−^) cells were Western blotted to analyze the subcellular distribution of p53. Tubulin and Histone-3 have been used as cytosolic and nuclear loading control. Data are representative of three independent experiments. (**e**) Percent cell death in p53-null cells upon over-expression of wild-type/mutant p53. Data are representative as the mean ± SEM and are the cumulative results of five independent experiments, *p < 0.05, **p < 0.01, ***p < 0.001. (**f**) Schematic diagram showing the basis of altered subcellular localization of mutant p53. Colored arrows lengths indicate the respective degree of nuclear localization of mutant p53.

Confocal imaging studies revealed that both p53WT and p53R273H shuttled into the nucleus (Fig. 5a, *first* & *second panel*) and colocalized with G13R-actin close to the perinuclear region [Pearson’s r = (+)0.60 and (+)0.744 respectively]. Co-immunoprecipitation assay confirmed physical interaction of p53R273H with G13R-actin (Fig. 5b). Zinc-binding domain mutant p53R175H, on the contrary, has been found to co-aggregate with other tumor suppressors, p63, and p73, showing cytosolic sequestration^12^. In wild-type p53 structure, Arg175 lies in the proximity of zinc-ion binding domain (Cys176, His179, Cys238 and Cys242), highlighted in (Fig. 5a, *third panel, right; PDB code: 2OCJ*)^29^. The introduction of the bulky imidazole ring (in His175) in place of p53Arg175 (R175H) contributes to loss of this tetrahedral coordination of Zn^2+^ ion^29^. Upon co-expression of p53R175H and G13R-actin-EGFP, p53R175H showed a punctate pattern of “cytoplasmic spots” near the perinucleus, and failed to translocate into the nucleus (Fig. 5a, *third panel*). Another structural mutant p53R249S shows a tendency to get accumulated in the cytoplasm^30^. Transient transfection of p53-null cells with p53R249S and G13R-actin revealed reduced colocalization between p53 and G-actin, suggesting towards their reduced physical interaction (Fig. 5a, *bottom panel*).

Next, we conducted co-immunoprecipitation experiments to assess the extent of G-actin association with p53 mutants and its putative role in nuclear localization. We found that all three p53 mutants exhibited a varying degree of interaction with G-actin, among which the interaction was more prominent for p53R273H (Fig. 5c). In case of other two structural mutants (R175H and R249S), a reduced affinity towards G-actin was observed. Structural distortion in the Zn^2+^ binding domain in p53R175H resulted in a reduced degree of p53:G-actin interaction. Likewise, the protein-protein association is significantly diminished in case of p53R249S mutant. This hints that Arg249 must be a residue participating in the interaction. This information was used as input for p53:G-actin docking calculation where Arg249 was considered as a constraint (Fig. 3d). We tried to correlate the varying degree of p53:G-actin interaction, with the respective subcellular localization of wild-type and mutant p53. Subcellular fractionation data revealed predominant nuclear localization of p53WT/R273H. Structural mutants’ p53R175H and p53R249S, on the other hand, were mostly found in the cytosolic fraction, which was indicative of their reduced nuclear import (Fig. 5d). Confocal studies revealed the same inference (Suppl. Fig. S7a,b). This diminished degree of nuclear translocation in case of p53R175H and R249S is attributed to their reduced affinity for G-actin to form p53R175H/R249S:G-actin complex. Taking together, these findings infer that the respective level of p53:G-actin interaction (in wild-type or mutant p53) dictates the subcellular localization of p53. Quantification of % cell death upon over-expression of p53 wild-type/mutants shows that mutant p53 bearing cells are more resistant to genotoxic stress exerted by Dox (Fig. 5e).

According to the p53:G-actin hypothetical model, p53Arg273 resides away from the most nearby residue (Glu152) of protein-protein interface (Fig. 5a, *second panel*). Hence mutation from p53Arg273 into His273 is not supposed to interfere with the p53:G-actin complexation. p53R73H forms a stable complex with G-actin and translocates into the nucleus. On the contrary, mutation at p53Arg249 into Serine, results in reduced p53:G-actin interaction. The simulation models for both p53wt:G-actin and p53R249S:G-actin were superimposed so as to find out the probable outcome of R249S mutation at the atomic level. Superimposition of post-simulated coordinates shows that in wild-type p53:G-actin complex, Arg249 (p53wt) forms a polar contact with Arg357 (G-actin). When mutated to Ser249, this crucial interaction is perturbed, whereas Arg357 forms another contact with p53Arg174. Hence the loss of Arg249-Arg357 interaction is compensated with the Arg174-Arg357 interaction. This change at the atomic level of p53:G-actin interaction can be hypothesized to be the reason for reduced degree of p53R249S:G-actin association. The movie of MD simulation trajectories has been included in the supplementary information (Suppl. Movie S2).

## Discussion

The tumor suppressor p53 is a 393 amino acid long transcription factor that mediates the cellular outcome in response to DNA damage^31^. In response to genotoxic stress, wild-type p53 gets stabilized and translocates to nucleus^32^. On the contrary, mutant p53 lose its wild-type tumor suppressive functions and acquires new oncogenic properties^33^. The tuning of its altered structure in course of mutation and gain of its new oncogenic properties has been well studied in literature^3^,^34^.

Here we report that monomeric G-actin regulates p53 nucleo-cytoplasmic shuttling in response to genotoxic stress. G-actin interacts with wild-type p53 and directs p53 traffic in reaching the nucleus. Co-expression of wild-type p53 and G-actin (G13R mutant) plasmids suggested that p53 interacts with the monomeric form of actin. A distinctive perinuclear pattern of colocalization was evident between p53 and G-actin, which inferred that the route towards perinuclear translocation of p53 is mediated by virtue of its interaction with G-actin. Histidine-pull down experiment confirmed that the binary interaction between p53 and G-actin is a direct protein-protein interaction. Superimposition of actin:actin and p53:G-actin model reveals that a common interface of protomer-3 of G-actin is involved in both the complex formation. This might account for reduced affinity of p53 towards F-actin. Interaction of p53 with G-actin is, therefore, a pre-requisite for p53 in order to get transported into the nucleus. Prior stabilization of actin with Jasplakinolide treatment, results in reduced conversion of F-actin to G-actin, which is assumed to be the reason for reduced nuclear translocation of p53. On the contrary, actin depolymerizing/G-actin sequestering drugs exert no such hindrance. The experimental results altogether, suggests a mechanism where G-actin directly interacts with p53 and escorts it towards perinuclear region. It is again to recall that previous studies suggested the role of actin in p53 nuclear transport, but our study distinctively revealed the contribution of G-actin in the p53 transport event.

Regarding p53 mutants, reports suggest that globally denatured p53 mutants are found as cytoplasmic aggregates in the perinuclear region^35^. Since the cancer-associated conformational mutants reveal altered subcellular localization, our assumption is that there must be some dispute in the mutant p53 nuclear trafficking machinery, which could be attributed to reduced interaction between mutant p53 and G-actin. Next, we aimed to confer how the position of a point mutation in p53 structure affects complexation of p53 and G-actin^12^. We have selectively chosen p53R273H, R175H, and R249S, which falls among the most frequent ‘hot-spot’ p53 mutants. Integrating our experimental information with theoretical predictions we observed the following results:

- Wild-type p53 interacts with G-actin, shows perinuclear colocalization in response to genotoxic stress, and translocates to the nucleus.
- p53R273H interacts with G-actin and translocates to the nucleus. Since according to the hypothetical model Arg273 resides away from the interface, mutation of p53Arg273 does not seem to interfere with p53:G-actin complexation.
- p53R249S shows reduced interaction with G-actin. It fails to get transported into the nucleus and predominantly shows cytoplasmic localization. The model proposes that Arg249 of p53 forms polar interaction with Arg357 of G-actin. Since Arg-Arg pairings have been found to be very crucial in making protein structures thermodynamically stable^36^, our assumption is Arg249-Arg357 imparts stability to p53:G-actin complex. Mutation at Arg249 into serine results in cytoplasmic sequestration of mutant p53, due to loss of its interaction with Arg357 of G-actin.
- p53R175H shows reduced interaction with G-actin, forms perinuclear aggregates and fails to get imported into the nucleus. It involves a mutation in the Zn^2+^ ion-binding domain, resulting in distortion of the native p53 structure^13^.

The above mechanism accounts for the G-actin-mediated regulation of p53 localization that unifies poorly connected information regarding wild-type/mutant p53 localization and provides a new angle for the understanding of the same. In a nutshell, to prevent the aberrant proliferation of the tumor cells, p53 gets transported into the nucleus and activates cell-cycle checkpoint proteins; promote DNA repair, cellular senescence, and apoptosis. The failure of p53 structural mutants to restrict abnormal malignant growth mainly stems from their inability to get efficiently transported into the nucleus. Therefore the biological consequence of mutant p53 mis-localization/cytoplasmic retention becomes significant as it accelerates tumor cell proliferation, migration, and invasion with the loss of tumor suppressive activity.

In conclusion, the inferences drawn from our study confirm the role of G-actin in p53 nuclear transport event, though it was not completely explored whether G-actin only delivers p53 in the perinucleus; or it also dictates the final entry of p53 inside nucleus. A report by Liang and Clarke demonstrates that once the p53 cargo reaches the perinuclear region, it interacts with importin receptors, probably through recognition of one or more nuclear localization signal (NLS-I, NLS-II, NLS-III)^37^. The NLSs of p53 interact with the carrier hetero-complex, importin-α/β, which facilitates the entry of p53 inside the nucleus^38^. Our assumption is that, NLS-recognizing importin-α/β hetero-complex binds the p53 cargo and drives its nuclear transit as p53:G-actin complex reaches the perinucleus.

Overall the present study emphasizes on the role of G-actin in guiding p53 transport towards the nucleus, which needs further study to unveil the contribution of other protein transporters associated with this event. Parallel to G-actin mediated traffic, other interactions mediated by importin-α/β at the nuclear membrane, also need to be explored with an effort to explain mutant p53 cytoplasmic retention.

## Materials and Methods

### Cell culture

A549 (lung carcinoma) cell line was purchased from National Centre for Cell Sciences, Pune. Human colon carcinoma HCT116 (p53^+/+^/p53^−/−^) and lung Carcinoma cell line H1299 (p53^−/−^) were purchased from ATCC, USA. The cell lines were cultured in DMEM or RPMI, supplemented with 10% heat-inactivated fetal bovine serum (Lonza, USA), L-glutamine (2 mM), sodium pyruvate (100 μg/ml), non-essential amino acids (100 μM), streptomycin (100 μg/ml), and penicillin (50 U/ml; Himedia, India). Primary antibodies used are p53 (DO-1; Santa Cruz, USA), α-GFP (Cell signaling, USA), α-Tubulin (Thermo Scientific, USA), GAPDH and Histone-3 (Biobharati Lifescience, India). Precision plus protein dual color standards (Bio-Rad) was used to detect the molecular weight of proteins in SDS-PAGE. Cyt-D, Lat-B, and Jas purchased from Calbiochem, USA was used at 5 μM, 1 μM, and 50 nM concentration respectively followed by Dox (1 μM, Sigma, USA) for 45 minutes. We made working stocks of all drugs (dimethyl sulfoxide for Cyt-D, Lat-B, and Jas; sterile H_2_O for Dox).

### Plasmid constructs and transfection

Over-expression plasmids encoding p53wt, contact, and structural p53 mutants, pCMV-Neo-Bam-p53wt, p53R273H, p53R249S (Addgene, USA)^39^ and p53R175H^40^ were transiently transfected for localization experiment or immunoprecipitation assay. Actin plasmids, wt-actin-EGFP/ G13R-actin-EGFP^17^/mCherry-LifeAct^41^ were over-expressed for immunoprecipitation and/or imaging experiments. For co-immunoprecipitation and/or immunofluorescence experiment, co-transfection of 1μg pCMV-Neo-Bam-p53wt/p53R273H/p53R175H/p53R249S and wt-actin-EGFP/G13R-actin-EGFP/mCherry LifeAct was performed using 5 μl of Lipofectamine 2000 (Invitrogen, USA), following the product manual. The complex of DNA and Lipofectamine 2000 was prepared in 200 μl serum-free OPTIMEM (Invitrogen, USA) and the complex was incubated for 20 min before adding to cells. For Histidine pull-down assay, His6-p53(1–393) bacterial expression plasmid was expressed in BL21-DE3 cells.

### Immuno-precipitation & Western blot

For whole cell lysates, cells were homogenized in buffer (20mM Hepes pH 7.5, 10 mM KCl, 1.5 mM MgCl_2_, 1 mM Na-EDTA, 1 mM Na-EGTA, and 1 mM DTT) supplemented with protease and phosphatase cocktail inhibitor^42^. For determining interaction between two proteins, co-immunoprecipitation technique was employed. For co-immunoprecipitation between p53wt/R273H/R175H/R249S and wt-actin-EGFP/ G13R-actin-EGFP, 200 μg protein was immuno-precipitated. Immunocomplex was purified using anti-p53 (DO-1) antibody and protein A/G-Sepharose beads (Invitrogen, USA). The immunopurified protein was immunoblotted with anti-GFP antibody. Subcellular fractions were generated using nuclear and cytosolic extraction buffer.

### Isolation of G-actin from rabbit muscle’s acetone powder

Actin was purified from muscle acetone powder of rabbit purchased from Sigma-Aldrich (M6890)^43^. 180 mg of muscle acetone powder was homogenized in buffer G (2 mM Tris-HCL (pH 8.0), 200 uM ATP, 0.5 mM DTT, 0.1 mM CaCl_2_, 1 mM NaN3). The Slurry was centrifuged at 30,000 g for 30 min, 4 °C. Pellet (0.38 gm) was re-suspended in 1 ml buffer G. 2 M KCl, 2 M MgCl2 and KCl were subsequently added and homogenized at 4 °C. Supernatant was spun at 100000 g, 2 h, 4 °C. The pellet was rinsed with 50 μl buffer G. Pellet was re-suspended into 2 ml buffer G and dounce homogenized. Dounced slurry was then dialyzed. Dialyzed actin was spun at 100000 g, 2 h, 4 °C. Supernatant, the ultimate G-actin was collected in a dialysis bag and stored in dialyzing G-buffer at 4 °C.

### Histidine pull-down assay using purified proteins

To purify His6-p53, BL21(DE3) harboring pET15-p53 vector was cultured at 37 °C until reaching A600 = 0.6, induced with IPTG (0.5 mM) and grown for another 3 h at 30° 44. Bacterial pellets were lysed in bacterial lysis buffer (25 mM Tris, 500 mM NaCl, 1% Triton-X 100, 5 mM Imidazole, protease-inhibitor cocktail, pH 8.0). Following centrifugation the supernatant was recovered and incubated with Ni-NTA agarose resin, and His-tagged p53 (1–393) was purified using a gravity flow column. Ni-NTA beads (Qiagen) bound with His-tagged p53 was washed in wash buffer (Tris-NaCl buffer with 20 mM Imidazole, pH 8.0) and subsequently eluted in elution buffer (Tris-NaCl buffer with 250 mM Imidazole, pH 8.0). His-tagged purified p53 was dialyzed against Tris-buffer (without imidazole). Purified His6-p53 was incubated with Ni-NTA beads, to which purified monomeric actin was added and incubated at 4 °C for overnight. Purified complex was eluted in elution buffer (Tris-NaCl buffer with 250 mM Imidazole, pH 8.0). Elute was run in 10% SDS-PAGE, followed by coomassie staining to probe interacting proteins in the complex.

### Immunofluorescence

Cells were grown on coverslips and fixed with 3.7%(v/v) paraformaldehyde for 20 min at RT, followed by permeabilization with 0.05%(w/v) Triton-X-100 for 5 minutes. Next, cells were blocked with 3%(w/v) BSA in PBS for 2 h. Primary antibodies (1:100) were diluted in 1%BSA in PBS and incubated for overnight at 4 °C. Cells were stained with Alexa-488 phalloidin and/or Alexa-594 DnaSe1 (Invitrogen, USA) for staining F-actin and/or G-actin, respectively, according to the manufacturer’s protocol. Secondary antibodies used (rabbit anti-mouse Alexa-488/-546, Invitrogen), were diluted in 1%BSA and incubated for 2 h at 4 °C. Nucleus was stained with DAPI (1:10,000). Images were acquired in Olympus fluoview confocal microscope/BX61 using 63×/100× objective. Laser intensities and detector gains were maintained at the same level during all imaging sessions. Z-stack images with an average step size of 0.35 μ were captured and 3D-projections of each stack processed for background correction. Image calculations and colocalization analysis have been done using Olympus fluoview and ImageJ software.

### Molecular docking with benchmark studies and MD simulation of p53:G-actin Complex

Crystal structures (*PDB code: 4MZR for p53, fragment: 94-356 and 3HBT for G-actin*) were used for protein-protein docking. Different docking algorithms; ZDOCK version 3.0.2^20^, Schrödinger’s protein-protein module PIPER^21^ and ClusPro 2.0^24^ were used for molecular docking. ZDOCK employs fast Fourier transform (FFT) correlation-based method, which performs a systematic search in the six-dimensional space created by 3 rotational and 3 translational degrees of freedom^20^. It has consistently been among the best-performing algorithms in the Critical Assessment of Prediction of Interactions (CAPRI)^45^,^46^. The scoring function implemented in ZDOCK is a linear weighted sum of van der Waals attractive and repulsive energies, short- and long-range attractive and repulsive electrostatic energies, and desolvation^47^. PIPER has also been found to determine the top scoring entries in the most recent CAPRI blind assessment of protein-protein docking. PIPER uses FFT approach along with accurate pairwise potentials that greatly reduces the number of false positive poses^21^. The scoring function in PIPER is given as the sum of terms representing shape complementarity, electrostatic and desolvation contributions^21^. ClusPro is another fully-automated docking algorithm which ranks the poses according to their clustering properties^24^. All atoms MD simulation in the implicit solvent was performed in AMBER14^48^. Force field parameters for ATP^49^, Calcium ion^50^, and Magnesium ion^51^ were obtained from Brayce group web server. Protein was parameterized using FF14SB force field parameters^52^ and ions were parameterized with GAFF force field parameters^53^. The protein system was neutralized with 9 Na^+^ ions. The system was minimized for 500 of minimization cycles, first, 250 cycles using steepest descent minimization and next 250 cycles using conjugate gradient minimization. Energy minimized system was used further for production simulation of 50 ns. The parameters set for simulations are as follows: calculation was performed for each 2 fs time step, coordinates were recorded at each 10 ps time step, temperature was maintained at 300K using Langevin thermostat with the collision frequency of 1ps^−1^ 54,55. Generalized Born implicit solvent simulation method was adopted for this study^56^. The implicit solvent is computationally much faster calculating the forces driving in between protein-protein complex. VMD and PyMOL software were used for visualizing the simulated trajectory^57^,^58^.

To establish docking benchmark for delineating the robustness of our p53:G-actin model, we took crystal structure of the complex p53DBD with BP2 (*PDB code: 1YCS*). The ability of ZDOCK to reproduce crystal structures was evaluated with the following procedure. First, the guest (BP2) and host (p53DBD) molecules in the complex crystal structure (*1YCS*) were separated, and the guest structure was then docked back into the host structure by ZDOCK. Of many ZDOCK-generated guest-host complexes, only the top models with the strongest interaction energy (according to the energy scoring function) were compared to the corresponding crystal structure of the complex. In this comparison, the individual docked poses were superimposed on the crystal structure *1YCS*, and the root mean square deviation (RMSD) values for all atoms were calculated. RMSD is frequently used to measure the quality of reproduction of a crystallographic binding pose by a computational method, such as docking^59^. If the docking protocol is able to reproduce similar docking pose of a ligand with respect to the same ligand in the crystal structure, then it validates the docking algorithm. If the RMSD is <2.0 Å or 1.0 Å, the crystal structure of the complex can be said to be reproduced or accurately reproduced by the program^60^. Using the similar parameters, docking calculations were done in PIPER and ClusPro2.0 for both *1YCS* and p53:G-actin complex. Knowledge of interface residue was also included in docking calculation to increase the likelihood of specified interaction. Data from mutational studies provide critical information for interaction, i.e. the complex does not form if certain residues are mutated^61^. Based on the mutational data, we used Arg249(p53) as a constraint for p53:G-actin docking calculation (similar to Arg248/Met243 constraint in p53:BP2 docking study).

### Statistical analysis

Values are shown as the standard error of mean (SEM) except where otherwise indicated. Comparison of multiple experimental groups was performed by 2-way ANOVA followed by a post-hoc Bonferroni modification of multiple comparison t-test. Data were analyzed and, when appropriate, the significance of the differences between mean values was determined by a Student’s t-test. Results were considered significant at p ≤ 0.05.

## Additional Information

**How to cite this article**: Saha, T. *et al*. G-actin guides p53 nuclear transport: potential contribution of monomeric actin in altered localization of mutant p53. *Sci. Rep.* **6**, 32626; doi: 10.1038/srep32626 (2016).

## Supplementary Material

Supplementary Information

Supplementary Movie S1

Supplementary Movie S2
