# Supplementary material for: G-actin guides p53 nuclear transport: potential contribution of monomeric actin in altered localization of mutant p53
Source: Sci Rep. 2016 Sep 7;6:32626. doi: 10.1038/srep32626 (PMC5013524; doi:10.1038/srep32626)
Supplement: Supplementary Information [file srep32626-s1.pdf]

Research Article

G-actin guides p53 nuclear transport: potential contribution of monomeric actin in altered localization of mutant p53

Taniya Saha[1], Deblina Guha[1], Argha Manna[1], Abir Kumar Panda[1], Jyotsna Bhat[2], Subhrangsu Chatterjee[2], and Gaurisankar Sa[1]*

[1]*Division of Molecular Medicine, Bose Institute, P-1/12, CIT Scheme VII M, Kolkata-700054, India*

[2]*Division of Biophysics, Bose Institute, P-1/12, CIT Scheme VII M, Kolkata-700054, India*

Supplementary figures:

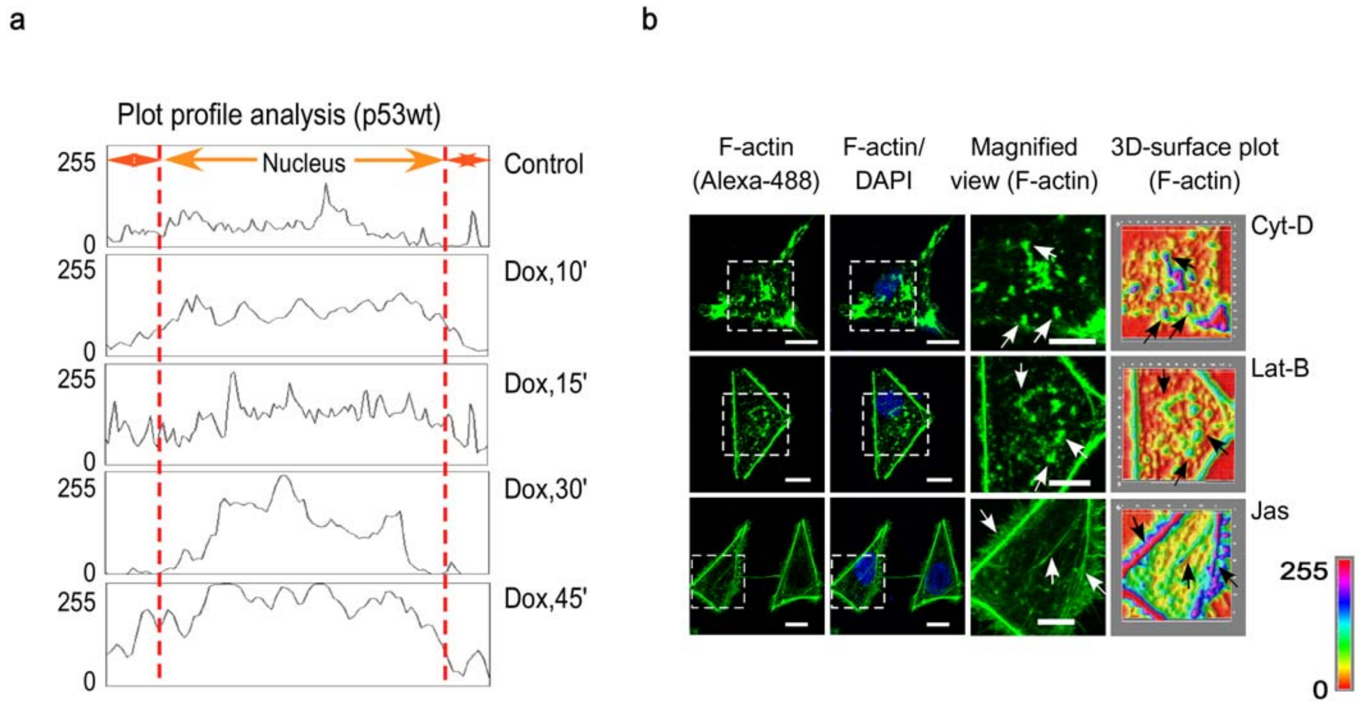

Supplementary Figure-S1: Nuclear distribution of p53. (**a**) Plot-profile analysis of p53 distribution at increasing time intervals of Dox treatment. White dashed line in the confocal images in (Fig. 1a) indicates the linear selection for plot-profile analysis. (**b**) Disruption of cytoskeletal actin structure in response to various actin-perturbing drugs, i.e. Cyt-D-/Lat-B-/Jas. White arrows in the magnified images indicate disrupted fibrous actin (Cyt-D and Lat-B) or stabilized actin fibers (Jas). 3D-surface plots of magnified images have been shown in the right panel (pixel intensity values 0 to 255).

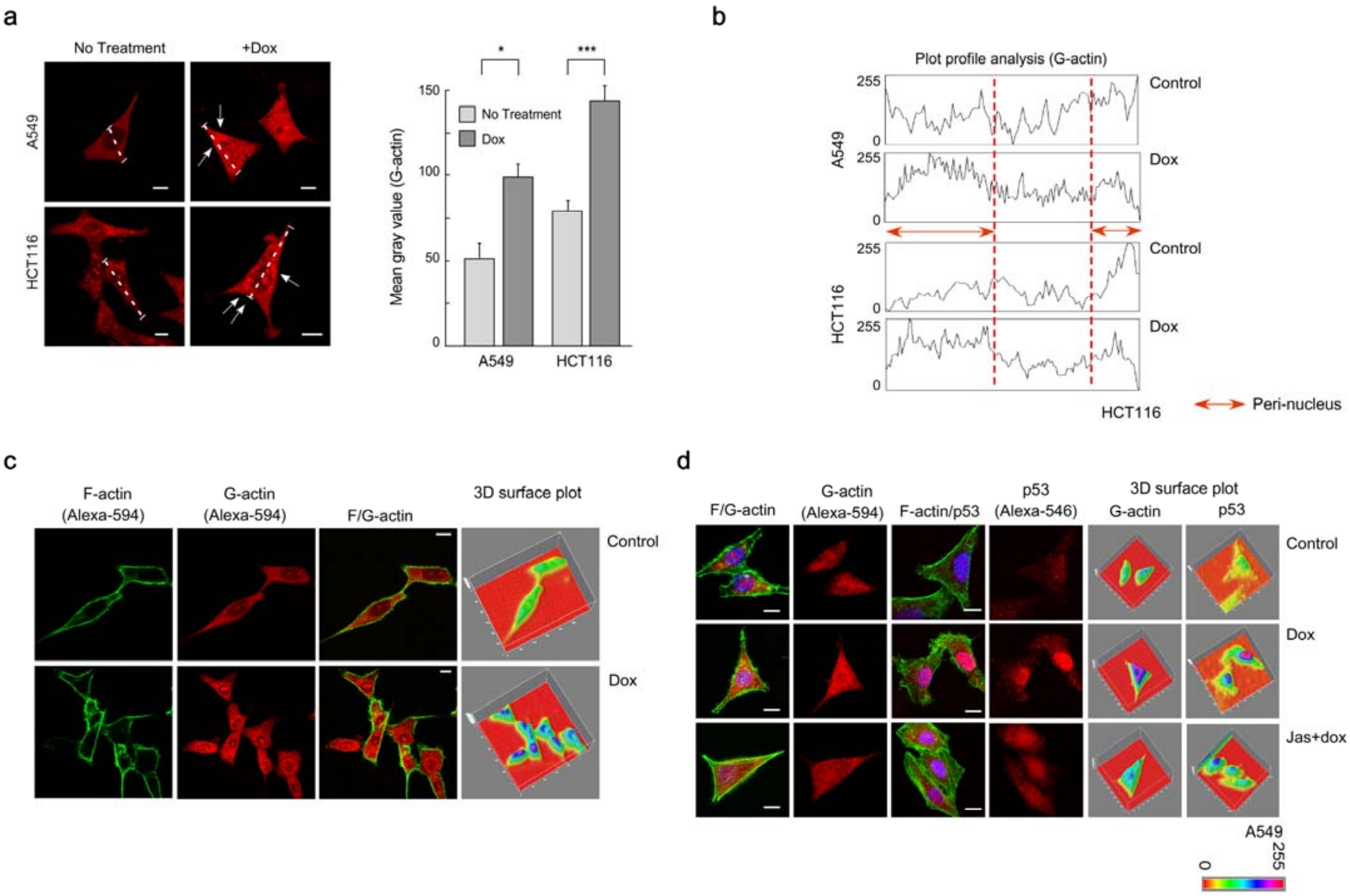

Supplementary Figure-S2: G-actin distribution in presence of Dox. A549 and HCT116 cells were treated with 1µM doxorubicin for 30′. (**a**) Confocal images showing level of G-actin (magnification 60x; scale bar: 10µM). Punctate distribution of G-actin is indicated by arrowheads. Mean gray value of G-actin distribution has been represented in right. (**b**) Plot profiles (mean gray value *vs* distance) of G-actin distribution. White dashed lines, in Fig. 2**a**, indicate the linear ROI selection (pixel intensity values 0 to 255). (**c**) F/G-actin distribution in HCT116 cells in control and doxorubicin-treated condition. F- and G-actin were stained with Alexa-488-tagged phalloidin and Alexa-594-tagged DnaSe1 respectively. (**d**) F/G-actin distribution and F-actin/p53 distribution in control, Dox, and Jas+Dox condition in A549 cells.

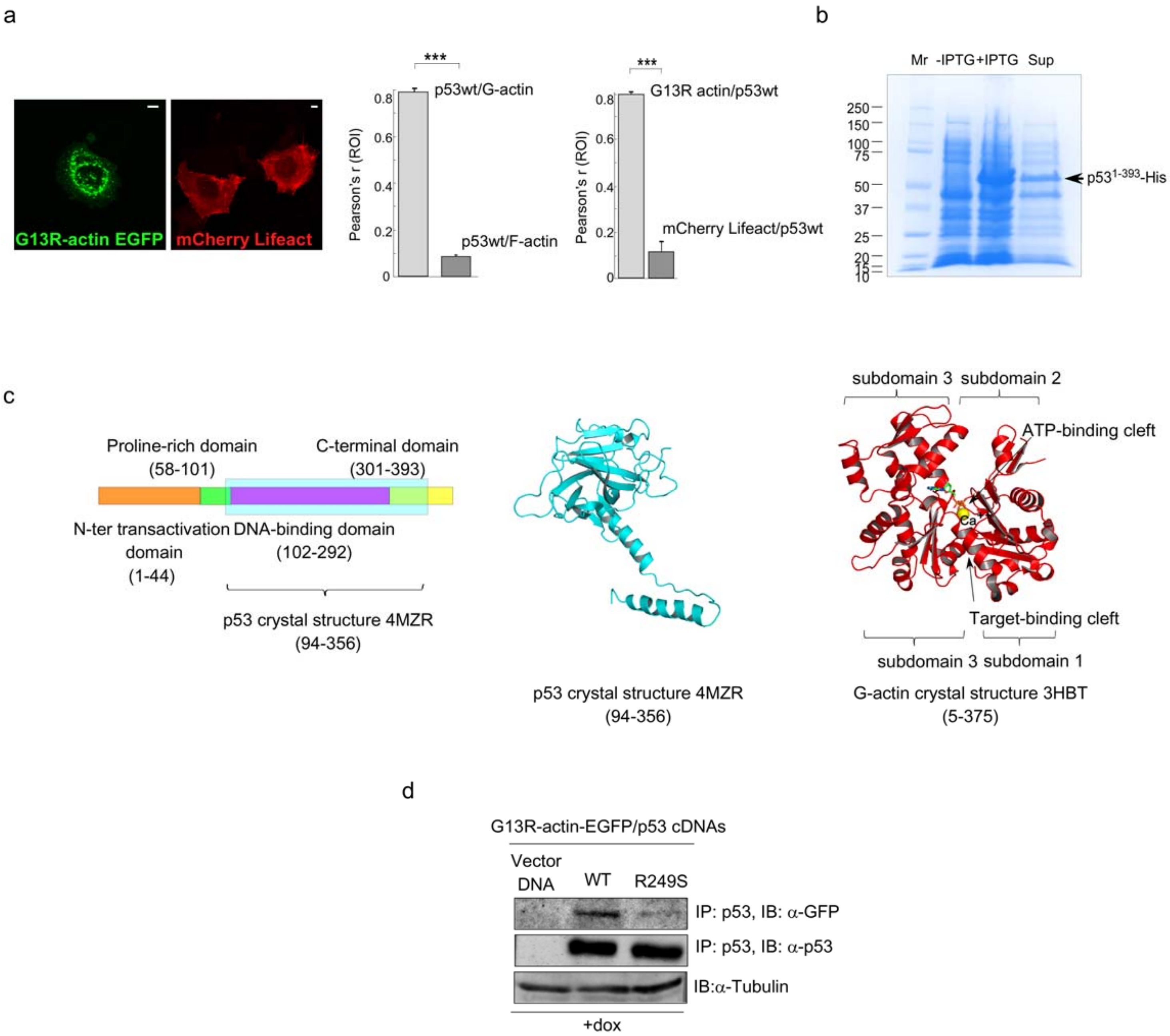

Supplementary Figure-S3: Domain structure of p53 and G-actin and the respective degree of colocalization correlation. (**a**) Expression of G13R-actin-EGFP and m-Cherry Lifeact, upon transient transfection of plasmids in H1299 cells (*left panel*); scale bar: 10μM. Bar diagram of Pearson's colocalization correlation between distribution of p53:G-actin and p53:F-actin (*middle*). Bar diagram of Pearson's colocalization correlation between distribution of p53:G13R-actin and p53:Lifeact (*right*). (**b**) Bacterial expression of His-p53 in absence or presence of IPTG, and in supernatant fraction indicated with arrowhead. (**c**) The schematic of domain structure of p53. Crystal structures of p53 (*PDB code:* 4MZR) and native G-actin structure (*PDB code:* 3HBT) are shown in right. (d) Physical interaction between G13R-actin and p53WT/R249S in p53-null H1299 cells, were performed by co-immunoprecipitation (with anti-p53 DO-1 antibody) and Western blot (with anti-GFP antibody) assays. *α*-Tubulin has been used as the internal loading control.

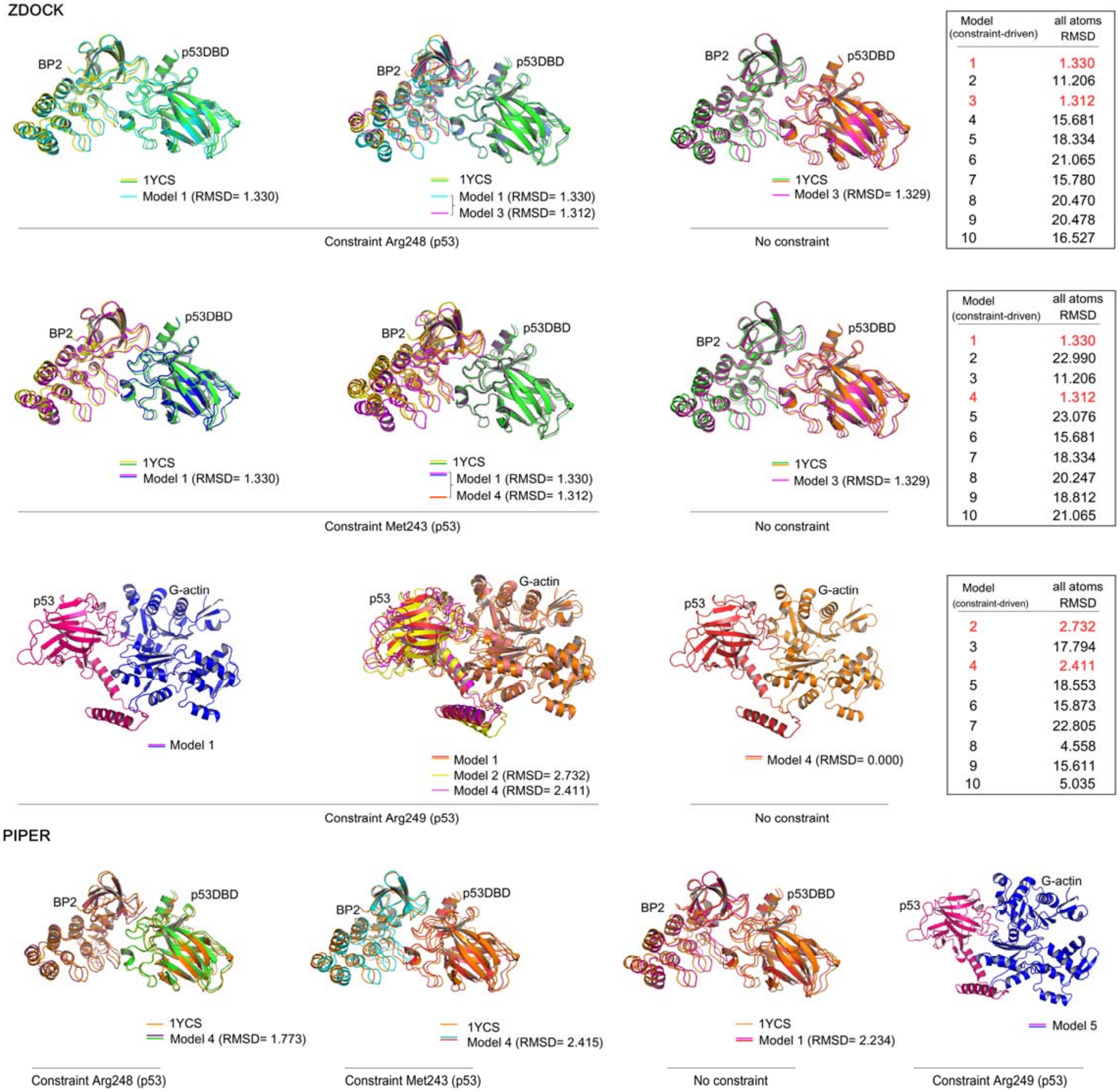

Model (constraint-driven)	all atoms RMSD
1	1.330
2	11.206
3	1.312
4	15.681
5	18.334
6	21.065
7	15.780
8	20.470
9	20.478
10	16.527

Model (constraint-driven)	all atoms RMSD
1	1.330
2	22.990
3	11.206
4	1.312
5	23.076
6	15.681
7	18.334
8	20.247
9	18.812
10	21.065

Model (constraint-driven)	all atoms RMSD
2	2.732
3	17.794
4	2.411
5	18.553
6	15.873
7	22.805
8	4.558
9	15.611
10	5.035

Supplementary Figure-S4: Docking benchmark studies in ZDOCK and PIPER using known crystal structure of p53DBD and BP2 complex (*PDB code:* 1YCS). Reproduced structures with/without docking constraint, Arg248 (*first panel*)/ Met243 (*second panel*), are shown. Reproduced p53:G-actin complex structures with/without docking constraint Arg249 (*third panel*). All atoms RMSD values with respect to *1YCS* (*first* and *second* panels) and Model 1 (*third panel*) are tabulated for each of the docking calculation. Results derived for *1YCS* and p53:G-actin complex using PIPER are shown in *fourth panel*. RMSD values of reproduced structures are highlighted in red.

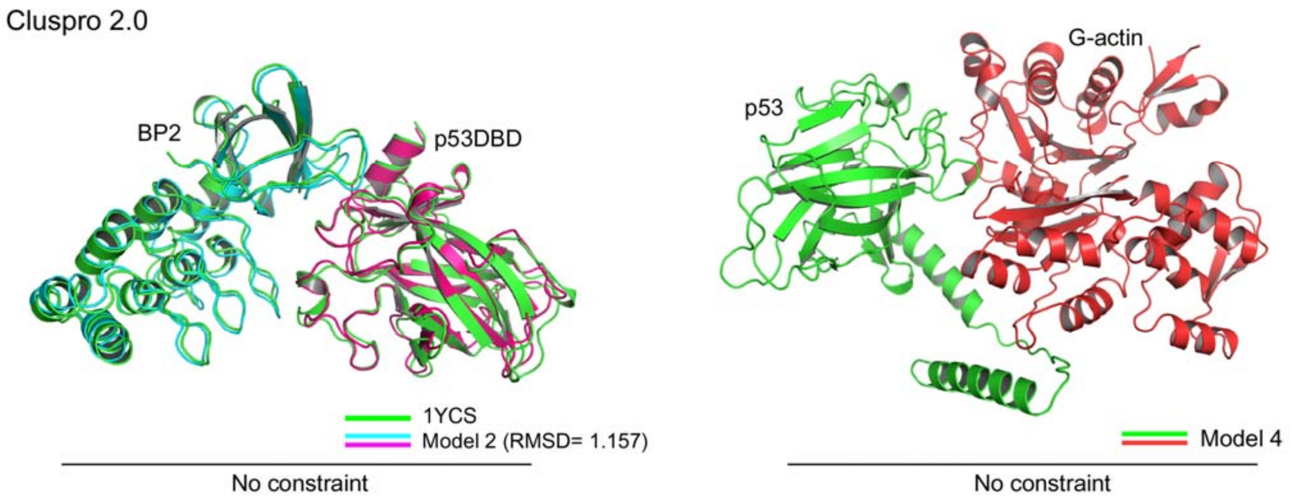

Supplementary Figure-S5: Docking benchmark studies in Cluspro 2.0 using known crystal structure of p53DBD and BP2 complex (*PDB code: 1YCS*). Reproduced structures for *1YCS (left)* and p53:G-actin (*right*), without docking constraint, are represented.

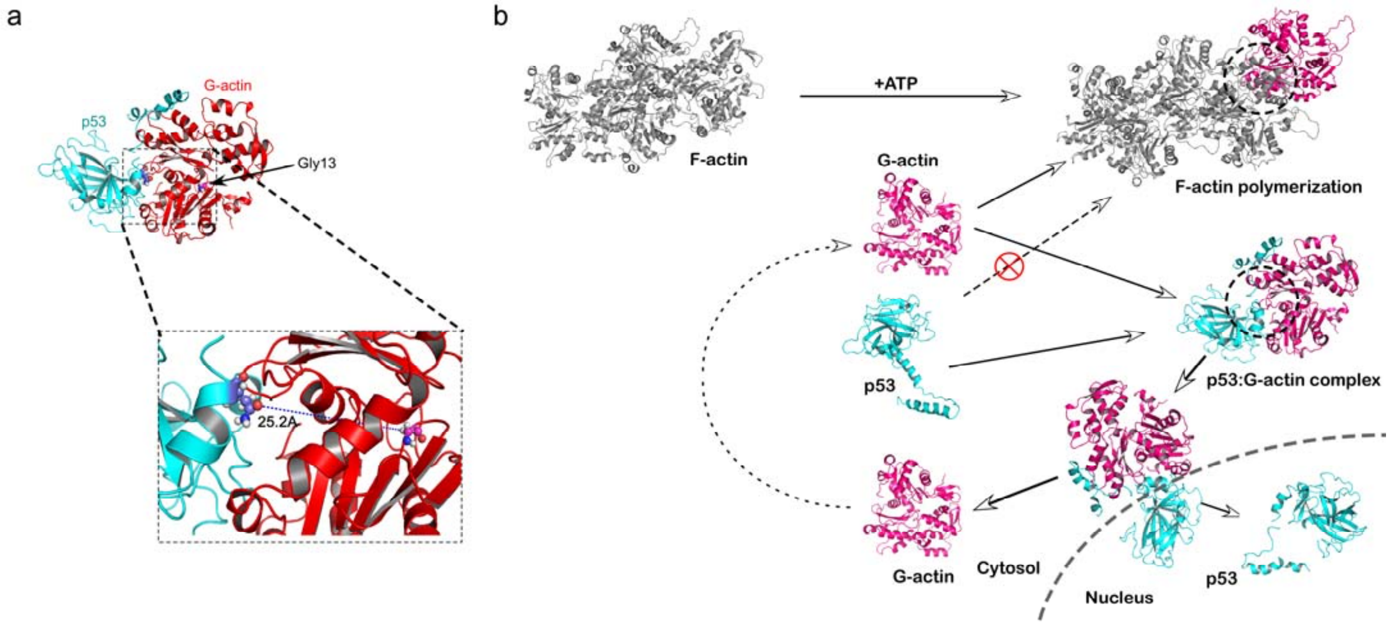

Supplementary Figure-S6: Schematic representation showing the basis of p53:G-actin interaction. (**a**) Ribbon-presentation of the post-simulation dynamic complex between wild-type p53 and G-actin, showing the position of Gly13 in G-actin. Magnified view is shown in the inset. (**b**) Ribbon structure depicting the formation of p53:G-actin complex in the cytosol, which is shuttled toward the nucleus, whereupon p53 dissociates from the complex and enters the nucleus. Since the site of p53:G-actin interaction overlaps with the region where monomer actin binds to the filament (marked with a black circle), F-actin retains poor affinity for p53.

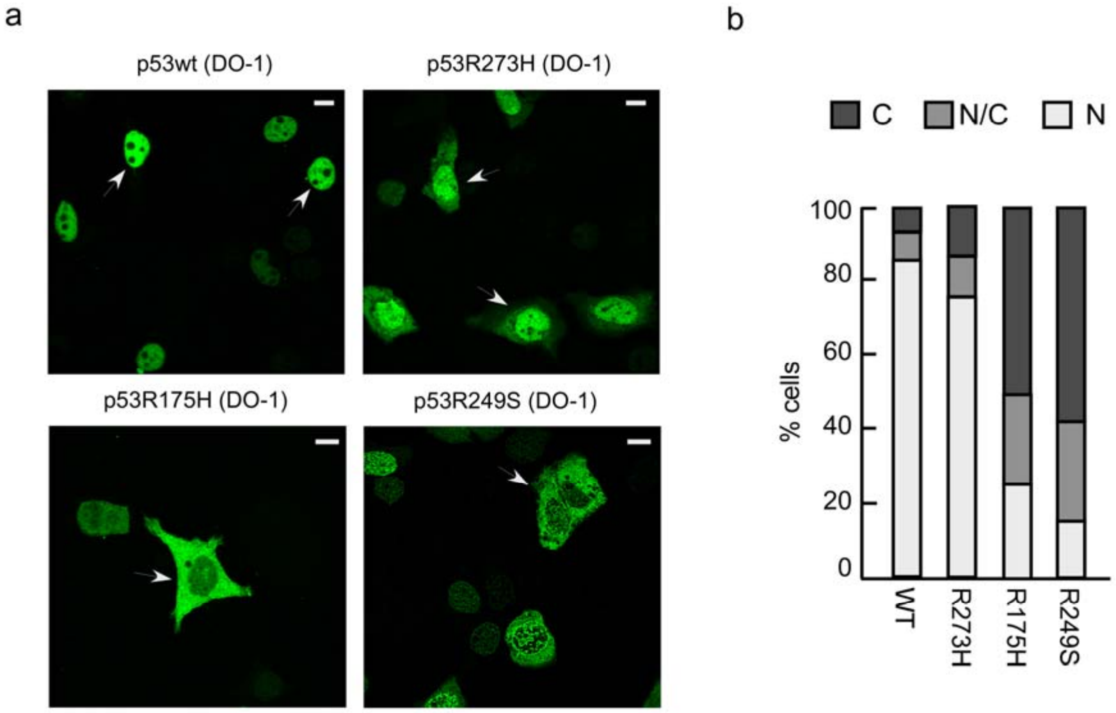

Supplementary Figure-S7: Subcellular localization of p53 wild-type/mutants. (**a**) Wild-type and mutant p53 were ectopically over-expressed in p53-null H1299 cells and stained with Alexa-488-tagged p53 (DO-1) antibody. Confocal imaging showing subcellular localization of p53; scale bar: 10μM. (**b**) Percentage cells showing nuclear, nuclear/cytoplasmic, and cytoplasmic p53 localization upon ectopically p53 over-expressed cells.

Supplementary Table-S1: The amino acid contacts revealed from p53:G-actin post-simulation dynamic complex are listed along with the predicted bond distance and interaction type.

p53 residues (dynamic complex)	G-actin residues (dynamic complex)	Predicted bond distance (Å)	Type of interaction
Gln165	Tyr151	2.4	Polar
His168	Tyr154	2.4	π -π interaction
Gln167	Ala155	2.5	Polar
Gly245	Arg357	3.3	Polar
Arg248	Glu152	2.1	Polar
Arg249	Arg357	1.8	Polar
Leu289	Glu152	2.5	Polar
Leu289	Thr133	2.8	Polar
His178	Ala350	3.9	Polar
Leu348	Ile272	2.9	Polar
Leu350	Ile272	2.7	Polar
Phe328	Thr309	2.8	Polar

Supplementary movie-S1: MD simulation trajectories (50ns) of the p53WT:G-actin complex in implicit solvent.

Supplementary movie-S2: MD simulation trajectories (50ns) of the p53R249S:G-actin complex in implicit solvent.
